# Perceptions of Registered Dietitian Nutritionists (RDNs) on the Use of Artificial Intelligence (AI) in Clinical Nutrition Care: A Cross-Sectional Survey Within a Large U.S. Healthcare System

**DOI:** 10.3390/nu18060934

**Published:** 2026-03-16

**Authors:** Danelle Johnson, Ryan T. Hurt, Manpreet S. Mundi, Bradley R. Salonen, Sara L. Bonnes, Darrell R. Schroeder, Shawn C. Fokken, Ivana T. Croghan, Jithinraj Edakkanambeth Varayil

**Affiliations:** 1 Division of Endocrinology, Diabetes, Metabolism and Nutrition, Mayo Clinic, Rochester, MN 55905, USA; 2 Department of Internal Medicine, Division of General Internal Medicine, Mayo Clinic, Rochester, MN 55905, USA; 3 Department of Biostatistics, Mayo Clinic, Rochester, MN 55905, USA; 4 Department of Family Medicine, Mayo Clinic, Rochester, MN 55905, USA

**Keywords:** artificial intelligence, nutrition, clinical nutrition, registered dietitian nutritionist

## Abstract

**Background:** Artificial intelligence (AI) is increasingly being integrated into healthcare, with applications ranging from predictive analytics to clinical decision support. In clinical nutrition, AI tools offer opportunities to improve workflow efficiency, enhance dietary assessment, and personalize nutrition care. Despite growing interest, little is known about registered dietitian nutritionists’ (RDNs) perceptions of AI in clinical practice. The aim of the present study was to assess RDNs’ attitudes toward AI use within a large healthcare system, along with their perceived barriers in this regard. **Methods:** A cross-sectional survey was developed through expert review and distributed electronically via REDCap to RDNs across Mayo Clinic’s academic campuses and affiliated health system sites. The 23-item survey included Likert-scale items addressing AI’s potential utilization within clinical care, perceived benefits and risks, and readiness for adoption. Responses were summarized using descriptive statistics. Factor analysis identified underlying constructs related to AI attitudes. Differences stratified by age and years of experience were evaluated using ANOVA. **Results:** Of the 185 RDNs invited, 64 (35%) responded. Two factors emerged: optimism regarding AI usage (Cronbach’s α = 0.94) and skepticism about implementation (α = 0.76). The overall mean ± SD score for optimism was 0.1 ± 0.6 (neutral), while skepticism averaged 1.0 ± 0.6 (moderate). Skepticism differed by years of experience (*p* = 0.012), with the lowest levels observed among RDNs with ≥21 years of practice. No significant differences were observed across age groups. **Discussion:** RDNs demonstrated neutral attitudes toward AI use but expressed concerns about accuracy, training, and implementation challenges. Addressing these concerns through education and structured implementation strategies may facilitate successful adoption of AI in dietetic practice.

## 1. Introduction

Artificial intelligence (AI) continues to rapidly transform healthcare [1]. AI assistants (AIAs) have been enhancing clinical workflows, supporting decision-making, and improving patient outcomes through predictive analytics, natural language processing (NLP), and machine learning (ML) applications [1,2]. AI’s role in healthcare spans several domains, including diagnosis, treatment, prognosis, clinician workflow, and the expansion of clinical expertise [3]. These technological advances are reshaping traditional care delivery models and prompting clinicians to reconsider how technology is integrated into their daily practice [1,2,3,4]. Registered dietitian nutritionists (RDNs) are central to translating nutrition science into actionable, individualized care plans and patient education. Yet many of the tasks that RDNs perform, such as creating tailored meal plans for complex medical conditions, accounting for cultural or religious preferences, and other patient-specific restrictions, are time consuming. In this context, AI may prove to be particularly relevant for dietetic practice by generating an initial draft, or educational materials that an RDN can efficiently review, personalize, and ensure to be clinically appropriate.

In clinical nutrition (CN), AI applications are increasingly being utilized to enhance dietary assessment, automate clinical documentation, and personalize nutrition recommendations [5,6,7,8]. These technologies have the potential to significantly improve the efficiency of dietetic consults, facilitate data analysis, and support patient education [8,9,10,11]. For example, in vision-based AI dietary assessment (VBDA), computer vision and deep learning techniques are used to analyze meal images for automatic detection of food type, portion size, and nutrient composition [12]. While earlier VBDA methods relied on multi-stage processes, recent advancements in deep learning have enabled end-to-end and multi-task learning models for more comprehensive dietary analysis [12]. Several smartphone-based applications have demonstrated feasibility and user satisfaction in dietary tracking, offering practical tools that can help both consumers and healthcare providers monitor eating behaviors [12,13,14]. In addition, various AI-based platforms can predict clinical outcomes and risks, such as malnutrition and disease onset, and aid in interventions by estimating nutrient intake and measuring postprandial glycemic response [15,16,17].

The introduction of new technology can bring various attitudes and perceptions to the surface, some of which could serve as barriers to implementation. Attitudes toward AI in healthcare vary across disciplines. However, there is a paucity of data about RDNs’ perceptions of the use of AI within U.S. healthcare systems. A recent study in which a survey was sent to dietitians in Saudi Arabia found that over half (62.7%) used AI in their practices. Younger dietitians were significantly more likely to have a higher level of knowledge of AI and possess a more positive attitude towards AI compared to their older counterparts. They found it to be helpful in creating personalized meal plans, education, and meal image analysis; however, they were concerned it might reduce personal interaction and the human touch to their work [18]. Another study surveyed dietetic students to assess their current use of generative artificial intelligence (GenAI) platforms, such as ChatGPT, and their perceptions of its future role in dietetic practice [19]. The majority of students were familiar with GenAI, with 52% of students reporting using it at least once a week, primarily for studying, generating ideas, and revising writing. They also believed that GenAI will be an integral part of future dietetics practice [19]. However, perceptions may differ between students and practicing clinicians, given differences in workflow demands, accountability, implementation burden, and prior exposure to AI. The purpose of the present study is to address this knowledge gap by describing RDNs’ perceptions, attitudes, and experiences related to AI during dietetic consults. We also conducted exploratory analyses of whether attitudes varied by respondents’ years of professional experience, age, and practice settings.

## 2. Methods

The present study was approved by the institutional review board of Mayo Clinic (IRB#: 25-001236). Members of a multi-disciplinary, outpatient nutrition support team including physicians and an RDN developed a 23-item survey. The survey included statements on general perceptions of AI, the role of AI in improving efficiency, trust in and the reliability of AI, patient engagement, outcomes, and ethical and practical considerations. Respondents rated their attitudes on a Likert scale. The survey focused on AI in general rather than specific GenAI applications.

Survey items were developed and refined through expert content review conducted by 10 subject matter experts (RDNs and physician nutrition experts), and minor revisions were made based on their feedback. This expert review supported face/content refinement but did not constitute a full psychometric validation. Construct validity, pilot testing with target respondents, and test–retest reliability were not assessed and are noted as limitations. This validation process has been used previously in a number of CN studies [20]. The survey also included an optional open-ended prompt to capture additional feedback; these responses were reviewed for context and to inform future survey refinement. The final survey was distributed electronically using REDCap (REDCap 15.0.10-© 2025 Vanderbilt University) (Research Electronic Data Capture) and sent out via email to all the RDNs within the Mayo Clinic health care enterprise who were working in a clinical capacity [21,22]. This included RDNs at Mayo Clinic Rochester, Florida, and Arizona. In addition to the three academic centers, the RDNs working in the larger Mayo Clinic Health System locations were sent the survey, which included southeast Minnesota, southwest Minnesota, northwest Wisconsin, and southwest Wisconsin. The survey remained open from 17 February 2025 to 7 March 2025. Participation was voluntary and anonymous.

## 3. Statistical Analysis

Respondent demographics were summarized using frequency counts and percentages. For analytical purposes, the responses to the 23 survey items assessing attitudes toward AI were coded as follows: −2 = strongly disagree, −1 = disagree, 0 = neutral, +1 = agree, and +2 = strongly agree. Centered Likert coding (−2 to +2) was used to facilitate interpretation of factor scores relative to neutrality. Responses to each item were summarized using frequency counts and percentages. An exploratory factor analysis was performed using promax rotation to allow for correlated factors. Based on an initial scree plot, a two-factor model was chosen. A factor loading cut-off of 0.40 was used to determine which items should be included in factor scores. Items with negative factor loadings were reversed, and Cronbach’s alpha was calculated for each factor to assess internal consistency. Factor scores were calculated using the average of the non-missing items, with a given factor score set to missing if ≥25% of the items were missing. Factor scores were summarized using means ± SDs and compared across categories of age and years of experience using analysis of variance (ANOVA), with *p* < 0.05 used to denote statistical significance. Given the modest sample size, factor analysis was performed for exploratory purposes only. Normality and homogeneity of variance were assessed visually; given the small subgroups, the ANOVA results were interpreted cautiously. Statistical analyses were performed using SAS version 9.4 (SAS Institute Inc., Cary, NC, USA).

## 4. Results

The survey was distributed to 185 RDNs within the Mayo Clinic enterprise, and 35% (n = 64) completed the survey. The respondents were proportionately spread across the age ranges from younger than 30 to older than 51 (Table 1); the greatest proportion of these RDNs (37%) had at least 21 years of work experience. The respondents were predominantly female (95%) and white (98%). Their work setting evenly reflected both inpatient and outpatient focuses, and they were allowed to select more than one option, as RDNs employed at smaller facilities may cover a combination of both areas.

Across the survey items, the respondents selected a range of responses for statements related to clinical efficiency, access to information, patient education, accuracy of AI tools, implementation costs, adequacy of training, and potential professional impact. Responses to the 23 Likert-scale items were analyzed using exploratory factor analysis, which identified two factors (Table 2). Factor 1 comprised 17 items, including one reverse-scored item (survey question #19), and Factor 2 comprised five items; one item (question #21) did not load onto either factor. The proportion of total variance explained was 63% for Factor 1 and 15% for factor 2. Internal consistency was high for Factor 1 (Cronbach’s α = 0.94) and acceptable for Factor 2 (Cronbach’s α = 0.76). No respondents were missing more than 25% of the items for either factor. Factor scores are summarized in Table 3. The overall mean ± SD score for Factor 1 was 0.1 ± 0.6, reflecting responses centered near the neutral point of the scale. The overall mean ± SD score for Factor 2 was 1.0 ± 0.6, indicating higher agreement with the items comprising this factor. Factor 1 scores did not differ significantly across age categories (*p* = 0.281) or years of experience (*p* = 0.642). Factor 2 scores did not differ significantly across age categories (*p* = 0.289); however, a statistically significant difference was observed across the category of years of experience (*p* = 0.012), with lower mean Factor 2 scores among the respondents with ≥21 years of professional experience (Table 3).

## 5. Discussion

This study provides insights into RDNs’ perceptions and attitudes toward AI use during dietetic consults within a large healthcare system. The findings indicate that the responses to survey items related to clinical efficiency, access to information, and patient education were largely neutral, whereas responses to implementation items showed greater agreement with skepticism-focused statements. Concerns also remain about its accuracy, the cost of adopting the technology, the ability to receive adequate training, and the overall impact of AI in the nutrition profession.

While we hypothesized that skepticism would be more prevalent among the most experienced RDNs in our survey population, the respondents with greater professional experience demonstrated lower scores on the implementation skepticism factor (Table 3). RDN respondents with fewer years of practice and younger respondents showed similar factor scores, without statistically significant differences according to age. These findings differ from some prior studies suggesting there are generational differences in technology acceptance across healthcare disciplines [23,24]. For example, a survey sent to pharmacists nationwide revealed that though approximately 50% of practicing pharmacists are aged 40 or younger, this group was much more familiar with AI and demonstrated higher AI usage rates than their more senior counterparts [24]. Younger professionals in healthcare are more likely to adopt new technologies, including AI. In the present study, factor scores did not differ significantly across age categories, and differences stratified by years of experience were limited to implementation-related items.

Examination of the survey items’ loadings for the identified factors provides additional insight into RDNs’ perceptions of AI. Across items, a substantial proportion of responses fell within the neutral category. Items reflecting skepticism largely centered on pragmatic concerns, including the financial costs of AI implementation and the availability of adequate training, suggesting apprehension related to the logistical feasibility of adoption. Additionally, the respondents expressed substantial concern regarding the potential for errors in AI-generated dietary recommendations and emphasized the importance of transparency and explainability in AI outputs, highlighting underlying issues of trust. In contrast, items associated with optimism emphasized the perceived ability of AI to streamline workflows, reduce time spent on routine tasks, improve productivity, and lessen overall workload. Items that loaded on the usage-related factor reflected agreement with statements describing workflow efficiency and clinical support. Despite these perceived benefits, respondents consistently indicated a need to conduct further research to better understand the role and impact of AI within dietetics. Taken together, the findings demonstrate neutral usage-related attitudes alongside implementation-related concerns. Additional studies are needed to clarify how AI adoption may ultimately affect RDN practice and the profession as a whole.

In comparison to other members of the multidisciplinary nutrition support team, these findings contribute discipline-specific data that can be compared with prior studies. Several studies have evaluated physicians’ perspectives on AI, often highlighting both optimism with respect to its potential and concerns about workflow disruption, ethical considerations, and loss of professional autonomy [1,18,19,20]. As with physicians, a few studies have investigated nurses’ perceptions of AI in healthcare, which have generally been positive [21]. Studies have reported that nurses are particularly comfortable with AI applications that assist in routine tasks, such as documentation and patient monitoring, as they view AI’s assistance as a way to spend more time with patients [22,23,24]. On the other hand, nurses have expressed apprehension regarding workload changes, patient safety, and the potential impact of AI on job roles [4,10]. Pharmacists have demonstrated optimism over AI-driven tools, particularly in regard to medication safety and error prevention, but have concerns about workflow, integration, and data privacy. A national survey of pharmacists in the United States found that although approximately 82% of pharmacists reported familiarity with AI, only 39% of them had experience using AI-based software [24]. While prior studies provide useful context, RDNs occupy a distinct role within healthcare that may shape their perception of AI. RDNs’ perceptions of AI may be shaped by profession-specific factors, including the highly patient-facing nature of dietetic consults, the emphasis on individualized counseling, and professional standards prioritizing clinical judgment and personalization.

Our study has several limitations. It was conducted within a single healthcare system, which may limit its generalizability to other settings. In addition, the respondents were homogenous in terms of race, ethnicity, and gender, which may further restrict the applicability of the findings to more diverse dietitian populations and limit the ability to assess variation in perceptions across demographic subgroups. Our response rate was low, resulting in a small sample size that may have reduced statistical power, particularly for subgroup analyses, and increased the potential for nonresponse bias, as individuals with greater familiarity with or stronger views on AI may have been more likely to participate. Additionally, while the survey included RDNs from multiple academic centers within the institution, the voluntary nature of participation introduces potential response bias. While we explored differences in attitudes based on age and years of experience, other factors, such as prior exposure to AI tools or digital literacy, may have influenced their responses and were not assessed in the present study, warranting future exploration. Finally, the survey assessed perceptions of AI in broad terms rather than focusing on specific AI applications or use cases. This lack of specificity may have introduced ambiguity in how the respondents interpreted AI use, potentially contributing to the high proportion of neutral responses across survey items and to variability in the interpretation of perceived benefits and concerns. Future studies are being designed to examine attitudes toward clearly defined AI tools and clinical applications to improve interpretability and relevance.

Despite these limitations, this study provides foundational data on RDNs’ attitudes toward AI and highlights both opportunities and challenges for its integration into CN practice. Future research should aim to validate these findings in larger, more diverse populations, including national organizations such as the Academy of Nutrition and Dietetics (AND) and the American Society for Parenteral and Enteral Nutrition (ASPEN). Longitudinal studies examining how education, training, and exposure to AI tools influence dietitians’ attitudes over time will also be valuable.

## 6. Conclusions

In conclusion, the findings indicate neutral attitudes toward AI use, along with moderate skepticism about its implementation. Tailored education, multidisciplinary collaboration, and ongoing research will be critical to support the effective integration of AI into nutrition care.

## Figures and Tables

**Table 1:** Demographic information on survey respondents (*n* = 64).

Characteristic	*n* (%)
Age, years	
≤30	14 (22%)
31 to 40	17 (27%)
41 to 50	17 (27%)
≥51	15 (24%)
Missing	1
Sex	
Female	59 (95%)
Male	3 (5%)
Missing	2
Years of experience	
≤5	14 (23%)
6 to 10	8 (13%)
11 to 15	10 (16%)
16 to 20	7 (11%)
≥21	23 (37%)
Missing	2
Race	
White	60 (98%)
More than 1 race	1 (2%)
Missing	3
Ethnicity	
Not Hispanic/Latino	59 (98%)
Hispanic/Latino	1 (2%)
Missing	4
Work Setting *	
Inpatient	33 (52%)
Outpatient	36 (56%)
Private Practice	1 (2%)
Memberships *	
Academy of Nutrition and Dietetics	21 (33%)
American Society for Nutrition	2 (3%)
American Society of Parenteral and Enteral Nutrition	11 (17%)
Other	11 (17%)

* Respondents could have reported multiple work settings and memberships. Note: When percentages were calculated, missing responses were excluded; denominators may vary by characteristic, and, due to rounding, the percentages may not sum to 100%.

**Table 2:** (**a**). Distribution of responses to survey items (*n* = 64). (**b**). Exploratory factor analysis loadings for survey items assessing attitudes toward artificial intelligence.

(**a**)						
**Item**	**Abbreviated Survey Statement**	**Strongly Disagree**	**Disagree**	**Neutral**	**Agree**	**Strongly Agree**
1	AI enhances consult quality	1 (2%)	4 (6%)	28 (44%)	24 (38%)	6 (10%)
2	The integration of AI aligns with the future of healthcare	1 (2%)	3 (5%)	14 (22%)	32 (51%)	13 (21%)
3	Confidence in AI’s accuracy	5 (8%)	17 (27%)	29 (46%)	12 (19%)	0 (0%)
4	AI addresses complex problems	4 (6%)	19 (31%)	21 (34%)	14 (23%)	4 (6%)
5	AI reduces time taken to complete routine tasks	1 (2%)	8 (13%)	7 (11%)	34 (53%)	14 (22%)
6	AI reduces workload	1 (2%)	7 (11%)	22 (34%)	25 (39%)	9 (14%)
7	AI can streamline personalized plans	2 (3%)	15 (23%)	19 (30%)	22 (34%)	6 (9%)
8	AI can improve productivity	1 (2%)	7 (11%)	24 (39%)	21 (34%)	9 (15%)
9	Dieticians trust AI recommendations	6 (9%)	16 (25%)	32 (50%)	10 (16%)	0 (0%)
10	AI provides evidence-based recommendations	4 (6%)	13 (20%)	39 (61%)	7 (11%)	1 (2%)
11	Clear explanations from AI are important	1 (2%)	0 (0%)	5 (8%)	34 (53%)	24 (38%)
12	Concern about AI’s errors	1 (2%)	4 (6%)	7 (11%)	32 (50%)	20 (31%)
13	Comfortable using AI with clinical judgment	3 (5%)	6 (9%)	17 (27%)	26 (41%)	12 (19%)
14	AI enhances patient engagement	3 (5%)	15 (23%)	34 (53%)	10 (16%)	2 (3%)
15	Patients are likely to trust AI advice	3 (5%)	22 (34%)	28 (44%)	11 (17%)	0 (0%)
16	AI improves patient outcomes	1 (2%)	12 (19%)	29 (46%)	19 (30%)	2 (3%)
17	Patients prefer AI-integrated consults	6 (9%)	23 (36%)	28 (44%)	7 (11%)	0 (0%)
18	AI improves communication and education	3 (5%)	9 (15%)	26 (42%)	22 (35%)	2 (3%)
19	The use of AI raises ethical/privacy concerns	1 (2%)	13 (20%)	19 (30%)	26 (41%)	5 (8%)
20	Cost is a barrier	2 (3%)	13 (21%)	37 (59%)	8 (13%)	3 (5%)
21	AI could replace dietitians in tasks	11 (17%)	26 (41%)	13 (20%)	12 (19%)	2 (3%)
22	Adequate training is necessary	1 (2%)	0 (0%)	6 (9%)	34 (53%)	23 (36%)
23	We need more research on AI	0 (0%)	0 (0%)	7 (11%)	32 (50%)	25 (39%)
(**b**)						
**Item**	**Abbreviated Survey Statement**				**Factor 1**	**Factor 2**
**1**	AI enhances consult quality				0.82	—
**2**	The integration of AI aligns with the future of healthcare				0.75	—
**3**	Confidence in AI’s accuracy				0.80	—
**4**	AI addresses complex problems				0.63	—
**5**	AI reduces time for routine tasks				0.80	—
**6**	AI reduces workload				0.86	—
**7**	AI can streamline personalized plans				0.86	—
**8**	AI can improve productivity				0.87	—
**9**	Dietitians trust AI recommendations				0.76	—
**10**	AI provides evidence-based recommendations				0.67	—
**11**	Clear explanations from AI important				—	0.74
**12**	Concern about AI’s errors				—	0.84
**13**	Comfortable using AI with clinical judgment				0.61	—
**14**	AI enhances patient engagement				0.65	—
**15**	Patients are likely to trust AI advice				0.55	—
**16**	AI improves patient outcomes				0.72	—
**17**	Patients prefer AI-integrated consults				0.46	—
**18**	AI improves communication and education				0.67	—
19 *	The use of AI raises ethical/privacy concerns				−0.50	—
**20**	Cost is a barrier				—	0.57
**21 ^†^**	AI could replace dietitians in tasks				—	—
**22**	Adequate training is necessary				—	0.70
**23**	We need more research on AI				—	0.45
	Cronbach’s Alpha				0.94	0.76

(**a**). Data were complete for all items except items 1, 2, 3, 16, and 20, which each had 1 missing response, and items 4, 8, and 18, which had 2 missing responses. Percentages may not sum to 100% due to rounding. (**b**). * Item 19 was reverse-scored when calculating the score for Factor 1 (optimism). ^†^ Item 21 did not load on either factor.

**Table 3:** Factor scores across age groups of members of the study population and their years of experience.

	Factor 1 (Usage Optimism)		Factor 2 (Implementation Skepticism)	
Characteristic	Mean ± SD	***p***-Value *	Mean ± SD	***p***-Value *
Overall (*n* = 64)	0.1 ± 0.6	-	1.0 ± 0.6	-
Age		0.281		0.289
≤30 (*n* = 14)	0.0 ± 0.8		0.9 ± 0.8	
31 to 40 (*n* = 17)	0.1 ± 0.6		1.1 ± 0.4	
41 to 50 (*n* = 17)	0.4 ± 0.6		1.0 ± 0.6	
≥51 (*n* = 15)	0.1 ± 0.5		0.8 ± 0.4	
Years of experience		0.642		0.012
≤10 (*n* = 22)	0.0 ± 0.7		1.0 ± 0.6	
11 to 20 (*n* = 17)	0.2 ± 0.6		1.3 ± 0.5	
≥21 (*n* = 23)	0.2 ± 0.6		0.7 ± 0.4	

Factor scores were compared across categories of the given characteristic using analysis of variance (ANOVA). * Item 19 was reverse-scored when calculating the score for Factor 1 (optimism).

## Data Availability

The original contributions presented in this study are included in the article. Further inquiries can be directed to the corresponding author.

## Abbreviations

RDN	Registered Dietician Nutritionist
AI	Artificial Intelligence
NLP	Natural Language Processing
ML	Machine Learning
RDN	Registered Dietitian
CN	Clinical Nutrition
VBDA	Vision-based AI Dietary Assessment
